# How Organic Mulching Influences the Soil Bacterial Community Structure and Function in Urban Forests

**DOI:** 10.3390/microorganisms12030520

**Published:** 2024-03-05

**Authors:** Wei Zhou, Xiangyang Sun, Suyan Li, Bingpeng Qu, Jianbing Zhang

**Affiliations:** 1 Key Laboratory for Silviculture and Conservation of Ministry of Education, College of Forestry, Beijing Forestry University, Beijing 100083, China; bsstzw@163.com (W.Z.); lisuyan@bjfu.edu.cn (S.L.); 2 Guangxi Key Laboratory of Earth Surface Processes and Intelligent Simulation, Nanning Normal University, Nanning 530000, China; zjb1166@163.com; 3 School of Environmental and Life Sciences, Nanning Normal University, Nanning 530000, China; qubingpeng@163.com

**Keywords:** organic mulching, soil bacterial community, urban forest

## Abstract

Urban forest soil is often disturbed by frequent human activity. Organic mulching is effective for improving soil quality; however, the effects of organic mulching on soil bacterial communities in urban forests are still largely unexplored. This study evaluated how organic mulching changed the urban forest soil bacterial community through an incubation experiment. Four treatments were applied: (1) no organic mulch (CK); (2) wood chips alone (5 g, Mw); (3) wood compost alone (5 g, Mc); and (4) wood chips + wood compost (This mulch was divided into two layers, i.e., the upper layer of wood chips (2.5 g) and the lower layer wood compost (2.5 g, Mw+c).) We found significant differences in the soil physicochemical properties under organic mulching after incubation. Overall, organic mulching can alter soil bacterial community structure. Soil alkali-hydrolyzable nitrogen, soil organic carbon, soil total nitrogen, and carbon-nitrogen ratio were the main factors affecting soil microbial community structures. Soil bacterial groups under organic mulching treatments mainly acted on the C and N cycling of functional pathways in soil. This study suggests that organic mulching could maintain the development of soil bacteria, which establishes a theoretical foundation for enhancing the microbiological environment of urban forest soils.

## 1. Introduction

Urban forests play a vital role in maintaining ecosystem stability and mitigating climate change [1]. Urban forest soil environments are also more complex than natural forests. Traditionally, frequent human activity (e.g., urban construction, environmental pollution, tourism development) has been a routine method of damaging urban forest soil quality. Further, unreasonable management leads to more serious soil degradation, consequently disrupting the equilibrium of the soil microbial community [2]. Therefore, regulating the urban forest soil has become an important ecological problem [3].

Urban forests and green spaces inevitably produce plenty of green waste by-products. Landfill storage and incineration of green wastes is a common practice for disposal of these residues, which causes serious environmental pollution and leads to accelerating soil nutrient loss. Therefore, how to utilize green waste effectively and eco-friendly becomes an important issue in China [4]. Organic mulching has been recommended for enhancing soil quality due to its capability to improve the soil environment [5], which can contribute to soil organic matter accumulation, reduce surface runoff, and increase soil water retention [6,7].

Organic mulches derive from many materials in agriculture and forestry (E.g., straw, wood chips, agriculture and forestry residues etc.). Wood chips, branches, leaves, and litter are the main mulch materials in forest ecosystems [8]. Furthermore, in order to accelerate the decomposition of forestry residues, wood composts as mulch materials were used to forest soil [9]. Organic mulches provide an available carbon source for soil microbial growth and alter the composition and diversity of microbial communities by improving the soil physicochemical properties [6,10]. However, most of the existing research focused on agricultural soils and mulches from urban forest waste (e.g., prunings, wood chips, strands), which have exceptional performance in urban forests. Nonetheless, their use for soil quality improvement is still unknown [11,12,13].

Soil microorganisms play an important role in the urban forest ecosystem by regulating material cycling and maintaining forest health [3,14,15]. Soil bacteria and fungi have become important indicators that reflect soil ecosystem multifunction [16]. Among the microbial communities, bacterial communities dominate the soil microenvironment and play a crucial role in decomposing organic matter in forests [17,18]. Soil microorganisms are sensitive to changes in soil physicochemical properties arising from different mulching models [6,19]. Organic mulching practices can improve soil microenvironment and directly affect the composition and diversity of microbial communities [6,19,20]. Understanding the effects of organic mulching on urban forest soil microbial communities is essential for the effective and sustainable management of urban ecosystems. Organic mulches from the urban forest wastes can be made into wood chips and wood compost for application in urban forests [9]. Different organic mulches had different ecological effects on the stability of terrestrial ecosystems and on addressing the problem of poor soil quality in urban ecosystems [6,7,12,21]. Several studies have revealed that single mulching practices can affect soil microbial community structure [22]. However, there is still a research gap underlying how different organic mulching models affect the soil bacterial community structure and function in an urban forest ecosystem.

It is necessary to demonstrate whether the soil bacterial communities are affected by different organic mulching models. Hence, we selected the urban forest soil of Beijing as a representative system, and used a 182 d incubation experiment and 16S rRNA gene sequencing to characterize the differences in the bacterial communities in urban forest soils with different mulching models. The following hypotheses are proposed: (1) organic mulching can change soil bacterial community structure and function by improving soil physicochemical properties; and (2) soil organic carbon and nitrogen, rather than other soil physicochemical properties, are the key factors that affect the changes in bacterial community structure and functional groups. The overall goal of this research study was to improve soil quality and establish a scientific and theoretical foundation for urban forest ecosystems.

## 2. Materials and Methods

### 2.1. Collection and Analysis of Soil and Organic Mulches

The soil used in the incubation experiment was collected in an urban forest located in the Jiufeng National Forest Park (40°4′10″ N, 116°6′25″ E), Haidian District, Beijing, China. The organic mulches (wood chips and wood composts) were collected in Xiangshan Botanical Gardens, and both materials had come from the main tree species (i.e., *Sophora japonica*, *Populus tomentosa*, *Fraxinus chinensis* Roxb.).

The air-dried soil used for incubation was randomly collected from the topsoil (0–10 cm), which was further passed through a 0.25 mm mesh sieve after visible plant debris and stones were removed by hand. The air-dried soil, before being used for incubation, had the following basic physicochemical characteristics: pH, 7.03; soil organic carbon (SOC), 21.24 g/kg; total nitrogen (TN), 1.72 g/kg; alkali-hydrolyzable nitrogen (AN), 112.62 mg/kg; available phosphorus (AP), 5.66 mg/kg; available potassium (AK), 197.75 mg/kg.

The mulches were dried to a constant weight (at 60 °C), and then ground and crushed. The particle size of wood chips and compost was 1–2 cm and 0.2 cm, respectively. The initial organic carbon contents of wood chips were 484.98 g/kg, total nitrogen contents were 7.77 g/kg, and C/N was 62.42. The initial organic carbon contents of compost were 356.68 g/kg, total nitrogen contents were 18.88 g/kg, and C/N was 18.89.

### 2.2. Experimental Design

**Figure 1:**
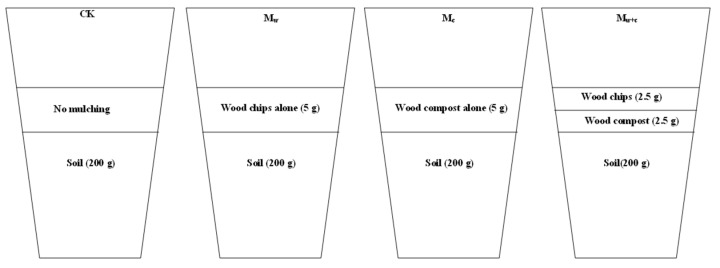
Schematic diagram of soil incubation.

Prior to incubation, the soil samples were sieved (0.25 mm) and air-dried. Next, the equivalent of 200 g dry soil was weighed into a 750 mL Mason jar. Four treatments were designed: (1) control (CK), no organic mulching on the soil; (2) wood chips alone (Mw), wood chips (5 g) were mulched on the soil; (3) wood compost alone (Mc), wood compost (5 g) was mulched on the soil; and (4) wood chips + wood compost (Mw+c); this mulch model was divided into two layers, i.e., the upper layer of wood chips (2.5 g) and the lower layer of wood compost (2.5 g) (Figure 1). Gauzes were used to separate soil and different mulches. The incubation temperature was 25 °C. Incubation lasted for 182 days, and all soil samples were moistened to 60% of water holding capacity by adding distilled water at regular intervals. Each sample was incubated in three replicates (n = 3) per incubation treatment. The samples for the destructive harvest were collected after 182 d.

### 2.3. Soil Physicochemical Properties Measurements

After incubation for 182 d, the soil was collected and air-dried to determine the physicochemical properties. The uppermost soil of the Mason jar has more contact area with organic mulches, so the uppermost soil was collected. The pH and electrical conductivity (EC) were measured using the pH and EC meter, respectively. SOC contents were determined using the potassium dichromate oxidation method. TN contents were determined using the Kjeldahl nitrogen determination method. AN contents were determined using the alkaline hydrolysis diffusion method. AP contents were determined using the molybdenum blue method. AK contents were measured with a flame photometer.

### 2.4. Soil DNA Extraction, Illumina Miseqsequencing

After incubation for 182 d, the fresh uppermost soil (0.5 g) in the Mason jar was collected to determine the soil bacterial community. In brief, total genome DNA was extracted using an OMEGA Soil DNA Kit (M5635-02) (Omega Bio-Tek, Norcross, GA, USA) and then checked on 0.8% agarose gel. The bacterial 16S rRNA gene (V3-V4 region) was amplified using primers 338F (5′-ACTCCTACGGGAGGCAGCA-3′) and 806R (5′-GGACTACHVGGGTWTCTAAT-3′). The purified PCR product of each sample was then pooled in equimolar concentrations in a single tube and sequenced using an Illumina MiSeq platform (Illumina MiSeq, Personalbio, Shanghai, China). Raw sequences were analyzed using the QIIME2 2019.4 and trimmed, and then quality filtered, denoised, merged and chimera removed using the DADA2 pipeline. Non-singleton amplicon sequence variants (ASVs) were aligned with mafft and used to construct a phylogeny with fasttree2. QIIME2 was used to calculate soil bacterial α-diversity indices (i.e., Shannon, Simpson, Chao1, phylogenetic diversity (Faith’s PD), and coverage). Soil bacterial β-diversity was calculated by principal coordinates analysis (PCoA), non-metric multidimensional scaling analysis (NMDS), and sample hierarchical clustering based on the Bray–Curtis distances. Linear discriminant analysis of effect size (LEfSe) was also employed to analyze the compositions of bacterial communities. In addition, we predict bacterial functions using the KEGG database in PICRUSt2 [23,24].

### 2.5. Statistical Analysis

Microsoft Excel 2019 was used to calculate the means and standard deviations of the raw data. A one-way analysis of variance (ANOVA) and Duncan multiple comparison test (*p* < 0.05) were used to analyze the significance of differences in soil physicochemical properties and bacterial diversity indices among the four treatments by SPSS 22.0 software. Furthermore, the bacterial community analysis and plotting of the results was performed in an online analysis platform (i.e., https://www.genescloud.cn, accessed on 1 December 2021).

## 3. Results

### 3.1. Effects of Organic Mulching on Soil Physicochemical Properties

**Figure 2:**
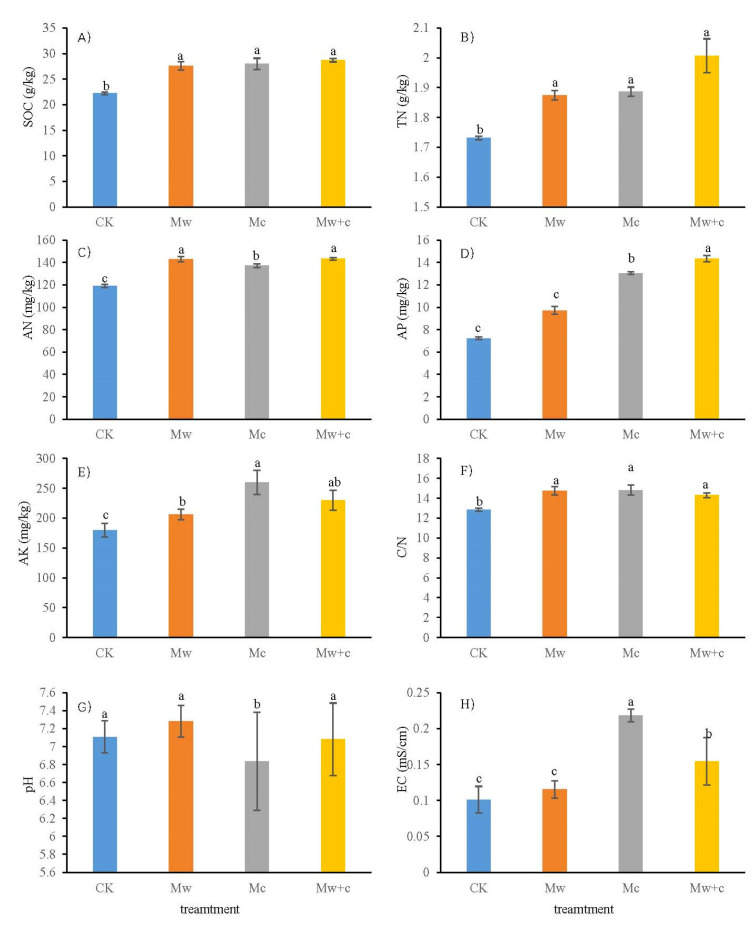
Characteristics of soil physicochemical properties under different treatments. (**A**) SOC, soil organic carbon; (**B**) TN, soil total nitrogen; (**C**) AN, alkali-hydrolyzable nitrogen; (**D**) AP, available phosphorus; (**E**) AK, available potassium; (**F**) C/N, carbon-nitrogen ratio; (**G**) pH, pondus hydrogenii; (**H**) EC, electrical conductivity. Lowercase letters indicate significant differences under different treatments (*p* < 0.05).

After a 182 d incubation period, compared with CK, SOC and TN contents were increased by 24.33–29.14% and 8.27–15.9% under the organic mulching treatments, respectively (*p* < 0.05; Figure 2A,B). Mw+c was higher than other treatments; however, no significant difference was observed among the different mulching models. Soil AN, AK, and C/N were also increased significantly in the organic mulching treatments (*p* < 0.05; Figure 2C,E,F). Soil pH was lower under the Mc treatment than under other treatments (*p* < 0.05; Figure 2G). In addition, AP and EC was lower under the CK treatment than Mc and Mw+c treatment (*p* < 0.05), while Mw treatment did not change significantly compared with CK (*p* > 0.05; Figure 2D,H). In short, organic mulching can improve soil C, N, and P contents.

### 3.2. Effects of Organic Mulching on Soil Bacterial Community

**Figure 3:**
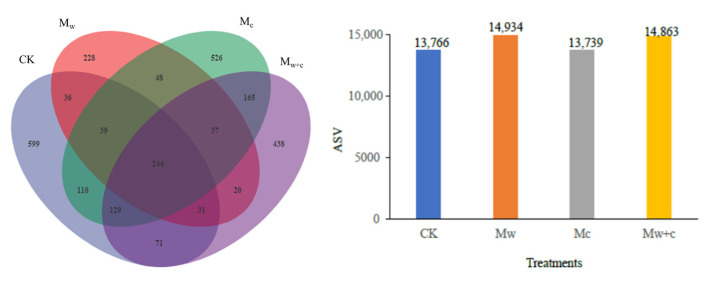
The Venn diagram of ASV number of soil bacterial under different treatments.

In total, 57,302 bacteria ASVs were obtained from 12 samples (Figure 3). The ASV distribution of soil bacteria in different treatments was Mw(14,934) > Mw+c(14,863) > CK(13,766) > Mc(13,739). No significant changes in the Simpson diversity index and coverage index were found in organic mulching treatments compared to CK (Table 1). However, the organic mulching treatments were found to significantly increase Shannon diversity index, Chao1 richness index, and PD index when compared to CK (*p* < 0.05; Table 1), suggesting that organic mulching can increase the diversity of soil bacterial community.

**Figure 4:**
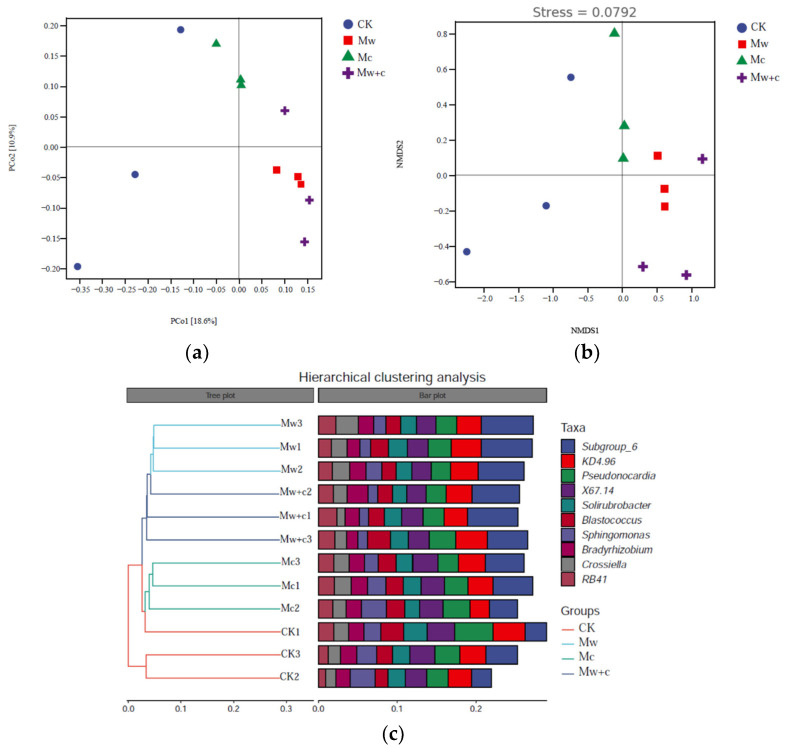
Principal coordinate (PCoA), Nonmetric Multidimensional scaling (NMDS), and hierarchical clustering analyses results of soil bacterial communities under different treatments. PCoA analysis of the bacterial community structure (**a**), NMDS analysis of the bacterial community structure (**b**), and Hierarchical clustering analysis (**c**).

PCoA and NMDS analysis revealed that bacterial β diversity was notably different between different treatments (Figure 4). The total variance explained by the first two axes (PCo1 and PCo2) were 18.6% and 10.9% of bacterial communities under the four treatments, respectively (Figure 4a). Based on the Bray–Curtis distance algorithm, hierarchical clustering trees at the ASV level of all samples demonstrated that the bacterial community structures could be classified into two groups, namely, the no wood chips treated group (CK and Mc) and wood chips treated group (Mw and Mw+c) (Figure 4c). ANOSIM tests of the Bray–Curtis analysis showed that the bacterial community structure was significantly different between different treatments (R^2^ = 0.56, *p* < 0.01; Figure 4b), which indicated that the bacterial community structure was changed under organic mulching.

As shown in Table 2, the relative abundances at the bacterial phylum level revealed that Actinobacteria (34.53−43.52%) was the most dominant phylum, followed by Proteobacteria (29.62−31%), Acidobacteria (7.94−14.03%), Chloroflex (7.14−9.8%), and Gemmatimonadetes (5.58−6.58%). The relative abundances at the bacterial class level revealed that Actinobacteria (19.3−27.32%) was the most dominant class, followed by Alphaproteobacteria (16.99−20.21%), Thermoleophilia (10.16−12.23%), Gammaproteobacteria (6.98−7.74%), and Gemmatimonadete (5.35−6.43%). The relative abundances of Actinobacteria were significantly enriched in CK compared with organic mulching treatments (*p* < 0.05). In contrast, the relative abundances of Acidobacteria were significantly enriched in organic mulching treatments compared with CK (*p* < 0.05). Organic mulching changed the relative abundances of the dominant bacteria (Actinobacteria and Acidobacteria), but different organic mulching models had no significant difference (*p* > 0.05).

**Figure 5:**
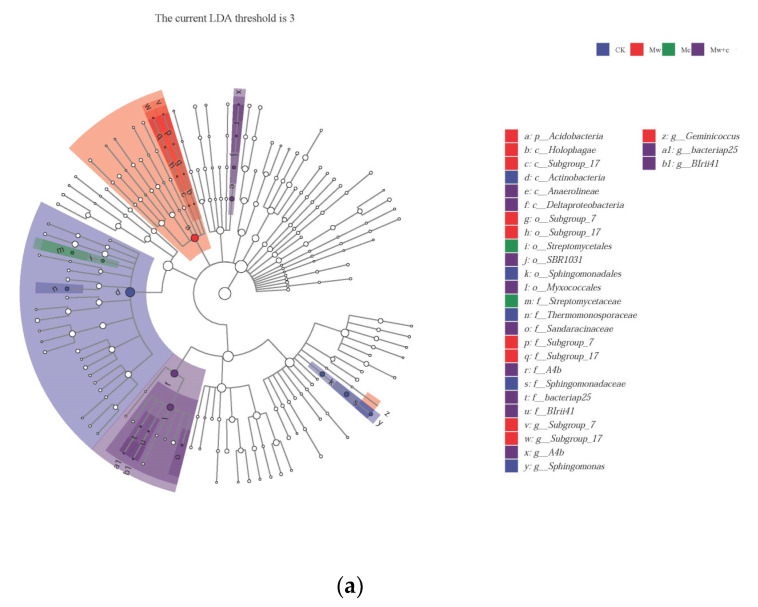

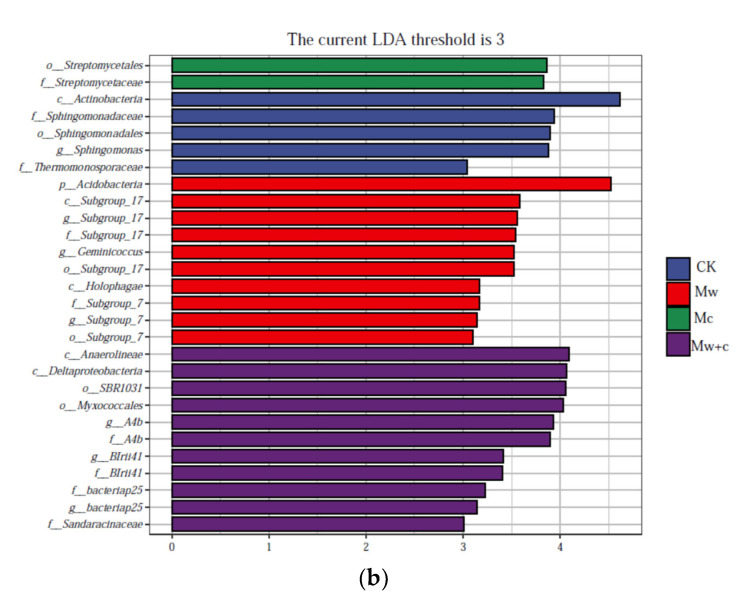
LEfSe analysis of soil bacterial communities under different organic mulching treatments. Cladograms showed the phylogenetic distribution of bacterial lineages associated with soils from the different treatments (**a**); Indicator bacteria with linear discriminant analysis (LDA) scores of 3 or greater in the bacterial communities under different treatments (**b**).

The taxa for each treated group were displayed in the cladogram (Figure 5). The indicator bacteria of the CK treatment were of the order Sphingomonadales (including family Sphingomonadaceae and genus *Sphingomonas*) and the class Actinobacteria (including family Thermomonosporaceae); those of the Mw treatment were of the phylum Acidobacteria (including class Holophagae and Subgroup-17) and genus *Geminicoccus*; those of the Mc treatment were belonging to the order Streptomycetales and family Streptomycetaceae; and those of the Mw+c treatment were of the class Anaerolineae (including genus and family SBR1031.A4b and order SBR1031) and order Myxococcales (including classes Sandaracinaceae, BIrii41, and bacteriap25). LEfSe can reveal the specific bacterial community composition. The results further revealed significant differences in abundance among treatments, with most taxa of bacteria being mainly enriched in the Mc and Mw+c treatments.

### 3.3. Relationship of Soil Physicochemicaly Properties and Bacterial Community

**Figure 6:**
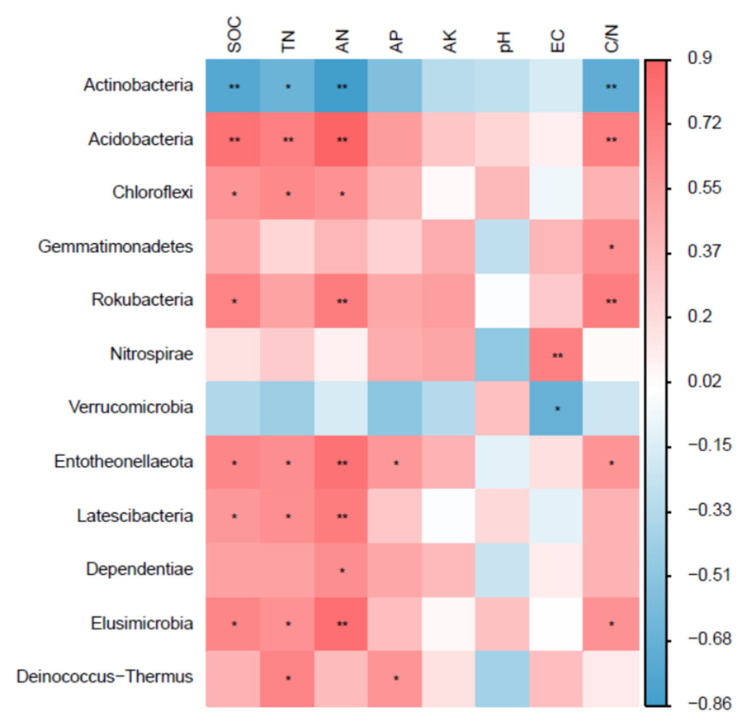
Correlation analysis between soil bacterial relative abundance (at phylum level) and soil physicochemical properties. *, *p* < 0.05; **, *p* < 0.01.

A Pearson’s correlation analysis is shown in Figure 6. The relative abundance of the phylum Actinobacteria was significantly or very significantly negatively correlated with SOC, TN, AN, and C/N, and the correlation coefficients were −0.76, −0.65, −0.86, and −0.72, respectively. The relative abundance of the phylum Acidobacteria was significantly or very significantly positively correlated with SOC, TN, AN, and C/N, and the correlation coefficients were 0.81, 0.73, 0.90, and 0.73, respectively. The relative abundance of the phylum Chloroflexi was significantly or very significantly positively correlated with SOC, TN, and AN, and the correlation coefficients were 0.61, 0.66, and 0.62, respectively. The relative abundance of the phylum Gemmatimonadetes was significantly positively correlated with C/N, and the correlation coefficient was 0.63. The relative abundance of the phylum Rokubacteria was significantly or very significantly positively correlated with SOC, AN, and C/N, and the correlation coefficients were 0.71, 0.74, and 0.73, respectively. The relative abundance of the phylum Nitrospirae was significantly positively correlated with EC, and the correlation coefficient was 0.73. The relative abundance of the phylum Verrucomicrobia was significantly negatively correlated with EC, and the correlation coefficient was −0.66. The relative abundance of the phylum Entotheonellaeota was significantly or very significantly positively correlated with SOC, TN, AN, AP, and C/N, and the correlation coefficients were 0.68, 0.66, 0.80, 0.59, and 0.60, respectively. The relative abundance of the phylum Latescibacteria was significantly or very significantly positively correlated with SOC, TN, and AN, and the correlation coefficients were 0.58, 0.64, and 0.74, respectively. The relative abundance of the phylum Dependentiae was significantly positively correlated with AN, and the correlation coefficient was 0.65. The relative abundance of the phylum Elusimicrobia was significantly or very significantly positively correlated with SOC, TN, AN, and C/N, and the correlation coefficients were 0.69, 0.62, 0.82, and 0.62, respectively. The relative abundance of the phylum Deinococcus-Thermus was significantly positively correlated with TN and AP, and the correlation coefficients were 0.69 and 0.60, respectively. The Pearson’s correlation analysis has shown that soil physicochemical properties (especially C and N) were closely correlated with bacterial community structures.

**Figure 7:**
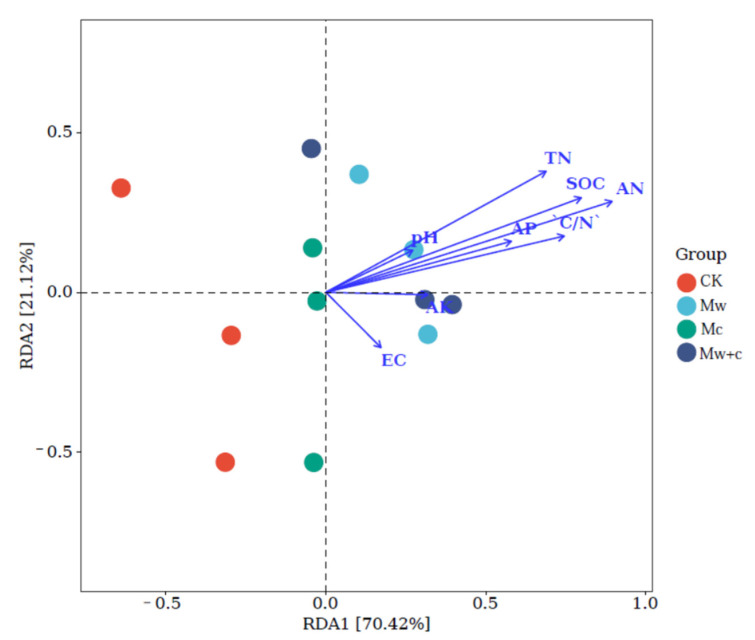
The RDA analysis between soil bacterial relative abundance (at phylum level) and soil physicochemical properties.

The RDA was conducted to explore the correlations between soil bacterial communities at the phylum level and soil physicochemical properties (Figure 7). The first two axes of the RDA model accounted for 93.48% of the total variance in the bacterial communities. Bacterial community composition was most highly correlated with soil AN (R^2^ = 0.84, *p* < 0.01), followed closely by SOC (R^2^ = 0.69, *p* <0.05), TN (R^2^ = 0.58, *p* < 0.05), and C/N (R^2^ = 0.56, *p* < 0.05). RDA analysis further demonstrated that C and N were the main factors affecting the soil bacterial community structures after organic mulching.

### 3.4. Effect of Organic Mulching on Soil Bacterial Community Function

**Figure 8:**
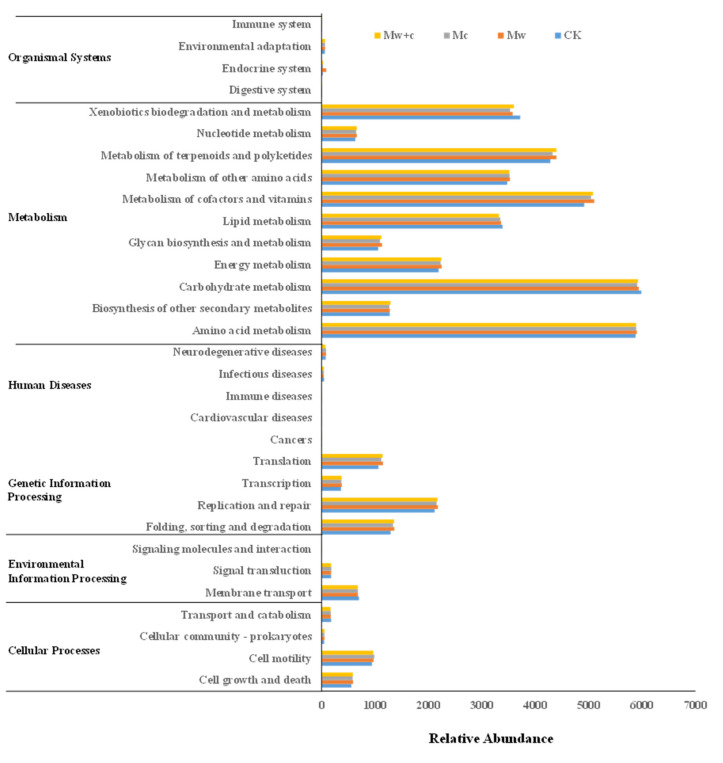
Abundance diagram of KEGG functional pathway for soil bacterial communities under different treatments.

Figure 8 shows the basic metabolic pathways, including cellular processes, environmental information processing, genetic information processing, metabolism, human diseases, and organismal systems. Compared to CK, the enrichment of genes related to Cell growth and death, Cellular community—prokaryotes, Folding, sorting and degradation, Translation, Immune diseases, Energy metabolism, Glycan biosynthesis and metabolism, Metabolism of cofactors and vitamins, and Nucleotide metabolism was significantly higher in the organic mulching treatments. The enrichment of genes related to Transport and catabolism, as well as Signaling molecules and interaction, was significantly lower in the organic mulching treatments than in CK. This observation indicates that organic mulching can affect soil bacterial function, but different organic mulching models did not show a significant difference. The KEGG annotation results suggested that metabolism was the most active functions. Metabolism is closely related to the carbon and nitrogen cycles.

## 4. Discussion

### 4.1. Organic Mulching Increased the Soil Nutrients

Organic mulching is an important practice for improving soil fertility and plant productivity in urban forest ecosystems. Our study revealed that organic mulching can significantly increase the soil nutrient contents (Figure 2), similar to the results of other mulching experiments [25,26]. This phenomenon may be mainly attributed to the decomposition of organic mulches, which can influence multiple soil properties and, through these changes, can indirectly affect soil microbial communities [3,12]. Numerous studies have demonstrated that mulching practice has beneficial effects on SOC and TN contents. We also found that organic mulching can increase SOC and TN contents, but different organic mulching models had no significant differences. This may be owed to the short incubation period of the experiment and the high content of C/N in woody material that cannot quickly decompose in a short timeframe. Zhao et al. [4] suggested that the addition of organic matters had favorable effects on soil fertility and productivity, and these effects became gradually positive over time. In addition, the beneficial effects depended on various conditions, such as the type of organic mulches, mulching thickness, and the soil properties [8]. In our previous research, after two years of mulching, Mw+c treatment remarkably increased the proportion of soil macro-aggregates, thereby increasing the soil aggregate stability [9]. Furthermore, the application of mulching organic materials increased SOC content, which might increase substrate level to promote bacterial growth and increase their abundance [27,28].

### 4.2. Organic Mulching Altered the Bacterial Community Structure and Composition

Organic mulching created an environment that is more conducive to the growth of beneficial microorganisms [29]. In general, organic material application improves soil characteristics and thus sustains a greater soil microbial diversity and community composition [23,24]. Maintaining high levels of soil bacterial diversity is very important for sustainable development of the ecosystem [4]. Many research studies have revealed that organic mulching can improve soil bacterial community structure and diversity [6,7,10]. This is similar to our current research findings. In this paper, the results revealed that organic mulching can increase α and β diversity of bacterial communities (Table 1, Figure 4). Organic mulching led to a slightly increased bacterial α-diversity but this has not yet reached a significant level. The results of β-diversity analysis based on Bray–Curtis distance matrix showed that the soil bacterial composition had significant differences between organic mulching treatments and CK. PCoA and cluster analysis results showed an obvious separation, indicating that organic mulches play an important role in bacterial community assembling [30].

Actinobacteria, Proteobacteria, and Acidobacteria were the most abundant phylum in the four treatments (Table 2), which was consistent with Yan et al. [31], who found that Actinobacteria was the most dominant phylum, followed by Proteobacteria and Acidobacteria under the biochar-based fertilizer amendments. Our study revealed that only Actinobacteria and Acidobacteria was remarkably different at the phylum level among CK and organic mulching treatments. Organic mulching treatments can increase the relative abundance of the phylum Acidobacteria and decrease Actinobacteria. At the class level, Blastocatellia (Subgroup_4), Acidobacteria, and others with Mw+c treatments were significantly different from CK. The phylum Acidobacteria is widely distributed in various ecosystems, which could play a role in decomposing stable and recalcitrant SOM; it was also associated with high SOM contents [32,33]. The concept of microbial nutrition strategies can help to understand the changes of soil bacterial community [16]. Members of the phylum Actinobacteria are suitable for managing a large variety of carbon sources, and played an important role in the biogeochemical cycling of soil carbon [31]. Actinobacteria are considered r-strategists, which thrive under copiotroph conditions. Conversely, Acidobacteria are known as K-strategists, and are relatively more abundant under resource-limited conditions. In this study, the relative abundances of K-strategists were significantly and positively correlated with soil nutrient contents, while the relative abundances of r-strategists had a negative relationship with soil nutrient availability. Acidobacteria is considered to have extensive metabolic and genetic functions [34]. Jiang et al. [16] pointed out that Acidobacteria had numerous subdivisions, and different subdivisions showed varied correlations with soil nutrients. Thus, we speculated that in the oligotrophic soil, organic mulching could especially stimulate the relative abundance of oligotrophic bacteria by increasing the available nutrient contents, which might help to improve soil productivity and sustainability in urban forest system [4].

Indicator microbes are the specialized communities that represent microbial communities with statistically significant differences [31]. Lefse analysis revealed that organic mulching significantly enriched biomarkers, and most taxa of bacteria were mainly enriched in the organic mulching treatments (Figure 5). Some biomarkers can improve soil quality and promote plant growth through phytostimulation, biofertilization, bioprotection, and other mechanisms [35].

### 4.3. Soil Bacterial Community Was Closely Related to Soil Physicochemical Properties

Understanding the relationship between soil bacterial community and soil physicochemical properties can help to develop strategies for ecological remediation [30]. Organic mulching can provide an ideal habitat for soil microorganisms and alter the soil ecosystem environment, which directly influences the bacterial community structure and function [30,36,37,38]. Exogenous nutrients play an important role in microbial community structure, which can change the composition of microbial communities [39]. Organic mulching increased soil nutrients and provided a microenvironment for microorganisms. In this study, soil physicochemical properties were closely related to soil bacterial community structure, and most soil nutrient factors had a negative correlation with Actinomyces and a positive correlation with Acidobacteria (Figure 6). This demonstrates that soil bacterial community structure was closely related to soil physicochemical properties, and the relationship was complex [39].

RDA indicated that the bacterial communities are closely related to the key soil physicochemical properties. The results showed that the AN, SOC, TN, and C/N were the main variables affecting bacterial community at the phylum level (Figure 7). This result indicated that soil C and N were the most important factors regulating bacterial communities, which was coherent with the previous research reported by Chi et al. [37]. Bacterial community changes depend on soil nutrient supply [16]. Several studies have demonstrated that soil physicochemical properties are crucial in shaping bacteria, and soil organic carbon and total nitrogen contents have been proven to be the predominant factors in determining soil microbial diversity [2,40]. Further evidence has shown that microorganisms are influenced by the ecological stoichiometric ratio (C:N:P) in forest ecosystems [17]. Organic mulching can significantly increase soil carbon and nitrogen contents, which provides carbon and nitrogen sources for soil bacteria and improves community structure.

### 4.4. Bacterial Function Was Altered under the Organic Mulching

The PICRUSt function can be used to predict the difference in bacterial communities’ metabolic pathways with organic mulching and provide potential phylogenetic resolution for bioremediation [30,37]. The changes in microbial communities may potentially affect the metabolic functional diversity [41]. In this study, the KEGG pathways of soil bacterial communities have obviously changed under organic mulching (Figure 8). Compared to CK, the abundance of the genes in some metabolic pathways (i.e., Cell growth and death, Cellular community—prokaryotes, Folding, sorting and degradation, Translation, Immune diseases, Energy metabolism, Glycan biosynthesis and metabolism, Metabolism of cofactors and vitamins, and Nucleotide metabolism) were increased by organic mulching, indicating that organic mulching exerted a strong positive effect on environmental adaptability, microbial growth, and energy metabolism [37]. The changes in soil microbial community structure can affect the pathways of essential metabolism [41]. Metabolism was the key pathway in microbial biogeochemical cycling. It is also beneficial to the carbon and nitrogen cycle [42]. Organic mulching changes soil physicochemical properties, increases soil moisture, and thus changes the biogeochemical cycling of C, N, and P [26]. Our research indicated that soil bacterial groups under organic mulching treatments mainly acted on the C and N cycling of functional pathways.

The abundance of most metabolism genes under organic mulching treatments was higher than those under CK, indicating that organic mulching can improve the metabolic activity of soil bacteria. Accordingly, organic mulching primarily altered the soil bacterial community structure and metabolic function. The main reason for the changed soil bacterial community structure and function may be ascribed to the shifts in soil nutrient contents. Cheng et al. [43] reviewed that soil nutrients can confer a notable effect on soil bacterial communities mainly via the shifts in resource acquisition and utilization. Therefore, organic mulching can directly regulate the soil microbial community or induce compositional shifts of microbes by changing soil physicochemical characteristics to improve soil quality. These findings have significant implications for understanding the distribution of the soil bacterial community after organic mulching in urban forest soil ecosystems. Nonetheless, our study was based on laboratory incubation experiment, so future field-based studies are needed to verify the impact of organic mulching on the soil bacterial community structure and function.

In brief, organic mulching can affect the soil micro-ecological environment. Soil bacterial community structure and composition under different organic mulching models were different. Our findings highlight the additive effects of organic mulching on soil bacterial communities, which enhanced the soil quality in an urban forest. However, thelong-term effects and mechanisms of organic mulching on soil microbial communities in urban forest are still unclear. Long-term monitoring systems must be established to research the effects of organic mulching on urban forests. In addition, incubation experiments cannot consider other contributing factors, such as climate, parent material, plant growth, land use, human activity, and other factors. In future, further research studies to reveal ecological mechanisms for soil microbial community are needed [36,44]. High-quality organic mulches can accelerate the turnover of bacterial biomass and the nutrient cycle [18]. Adding beneficial microorganisms to organic mulches will be more conducive to improving the soil quality [35].

## 5. Conclusions

Organic mulching had additive effects on the soil bacterial communities and soil properties in the urban forest. The contents of soil nutrient were higher in organic mulching treatments than CK, indicating that the application of organic mulches could improve soil quality. Organic mulching can also change soil bacterial community composition and structure. RDA showed that soil organic carbon and soil nitrogen were the most important soil factors to explain the bacterial community composition. Soil bacterial groups under organic mulching treatments mainly acted on the C and N cycling of soils of functional pathways. Long-term dynamic monitoring is needed to explore the ecological effect of organic mulching to provide a foundation for urban forest soil improvement.

## Figures and Tables

**Table 1:** The α-diversity indexes of soil bacterial communities under different treatments.

Treatments	Shannon	Simpson	Chao1	PD	Coverage (%)
CK	11.14 ± 0.21 b	0.998 ± 0.08 a	6273.48 ± 198.99 b	274.45 ± 6.23 b	98.02 ± 0.15 a
Mw	11.46 ± 0.02 a	0.999 ± 0.003 a	7080.63 ± 89.95 a	319.84 ± 9.95 a	97.59 ± 0.04 a
Mc	11.29 ± 0.23 ab	0.999 ± 0.05 a	6525.27 ± 628.96 a	286.99 ± 27.61 ab	97.78 ± 0.31 a
Mw+c	11.47 ± 0.12 a	0.999 ± 0.01 a	6814.58 ± 434.18 a	315.52 ± 14.10 ab	97.89 ± 0.41 a

Lowercase letters indicate significant differences under different treatments (*p* < 0.05).

**Table 2:** Relative abundances of soil bacterial communities under different treatments at phylum and class level.

Phylum	Relative Abundances (%)			
	CK	Mw	Mc	Mw+c
Actinobacteria	43.52 ± 3.32 a	34.53 ± 1.79 b	38.09 ± 0.14 ab	34.69 ± 3.48 b
Proteobacteria	29.90 ± 3.80 a	29.62 ± 2.33 a	31.00 ± 2.68 a	29.69 ± 2.70 a
Acidobacteria	7.94 ± 1.32 c	14.03 ± 0.74 a	11.12 ± 0.63 b	13.63 ± 1.31 ab
Chloroflexi	7.14 ± 0.19 a	9.10 ± 0.64 a	7.38 ± 1.75 a	9.80 ± 0.86 a
Gemmatimonadetes	5.58 ± 0.71 a	6.35 ± 0.14 a	6.58 ± 0.52 a	5.79 ± 0.30 a
Bacteroidetes	1.37 ± 0.75 a	1.31 ± 0.22 a	1.24 ± 0.19 a	1.47 ± 0.22 a
Rokubacteria	0.73 ± 0.10 b	0.98 ± 0.14 a	0.96 ± 0.02 ab	0.91 ± 0.08 ab
Patescibacteria	0.75 ± 0.07 a	0.94 ± 0.34 a	0.82 ± 0.25 a	0.91 ± 0.20 a
Firmicutes	0.96 ± 0.51 a	0.36 ± 0.40 a	0.67 ± 0.08 a	0.61 ± 0.13 a
Nitrospirae	0.54 ± 0.10 a	0.49 ± 0.06 a	0.65 ± 0.11 a	0.59 ± 0.06 a
Others	1.57 ± 0.227 a	2.30 ± 0.29 a	1.48 ± 0.36 a	1.90 ± 0.73 a
**Class**				
Actinobacteria	27.32 ± 1.16 a	19.30 ± 1.27 b	22.18 ± 1.49 b	19.88 ± 3.09 b
Alphaproteobacteria	20.21 ± 2.22 a	17.87 ± 2.50 a	19.27 ± 2.44 a	16.99 ± 2.58 a
Thermoleophilia	12.23 ± 1.90 a	10.93 ± 0.55 a	11.45 ± 1.17 a	10.16 ± 0.41 a
Gammaproteobacteria	6.98 ± 1.99 a	7.40 ± 0.42 a	7.74 ± 0.55 a	7.69 ± 0.34 a
Gemmatimonadetes	5.35 ± 0.72 a	6.28 ± 0.12 a	6.43 ± 0.52 a	5.70 ± 0.29 a
Subgroup-6	3.30 ± 0.88 c	6.618 ± 0.39 a	4.70 ± 0.82 bc	6.09 ± 0.74 ab
Deltaproteobacteria	2.68 ± 0.55 b	4.32 ± 0.84 a	3.96 ± 0.36 ab	4.97 ± 0.61 a
KD4-96	3.43 ± 0.57 a	3.47 ± 0.35 a	3.01 ± 0.48 a	3.44 ± 0.55 a
Blastocatellia-(Subgroup-4)	2.11 ± 0.54 b	2.81 ± 0.41 ab	2.85 ± 0.10 ab	3.11 ± 0.25 a
Acidobacteriia	1.91 ± 0.13 b	2.81 ± 0.24 ab	2.47 ± 0.45 ab	2.98 ± 0.54 a
Others	14.46 ± 0.94 c	18.19 ± 0.61 ab	15.94 ± 1.7 bc	18.99 ± 0.24 a

Lowercase letters indicate significant differences under different treatments (*p* < 0.05).

## Data Availability

Data are contained within the article.
